# Early cardiac changes following anthracycline chemotherapy in breast cancer: a prospective multi-centre study using advanced cardiac imaging and biochemical markers

**DOI:** 10.1186/1532-429X-14-S1-P181

**Published:** 2012-02-01

**Authors:** Suchi Grover, Carmine DePasquale, Darryl P Leong, Adhiraj Chakrabarty, Kerry A Cheong, Dusan Kotasek, Rohit Joshi, Amy Penhall, Lucas Jeorg, Majo Joseph, Bogda Koczwara, Joseph Selvanayagam

**Affiliations:** 1 Cardiology, Flinders Medical Centre, Adelaide, SA, Australia; 2 Oncology, Flinders Medical Centre, Adelaide, SA, Australia; 3 Flinders University, Adelaide, SA, Australia; 4 Adelaide Cancer Centre, Adelaide, SA, Australia; 5 Lyell McEwin Hospital, Adelaide, SA, Australia

## Summary

This prospective study is designed to identify novel imaging (utilizing cardiovascular magnetic resonance and advance echocardiography) and biochemical markers to detect early, sub-clinical cardiotoxicity following chemotherapy.

## Background

Cardiac toxicity is an important long term side effect of anthracyline chemotherapy. We hypothesized that novel cardiovascular magnetic resonance (CMR) and echocardiographic markers of myocardial function, oedema, and necrosis can detect early, subclinical cardiac toxicity in patients receiving anthracycline therapy for breast cancer.

## Methods

29 chemonaive patients with breast cancer (early stage and metastatic), underwent serial cardiovascular magnetic resonance imaging (for LV volumes/ejection fraction, myocardial oedema and necrosis), advanced echocardiography (for LV strain in addition to standard measurements of LV diastolic function), pro brain natriuretic peptide (pro-BNP) and high-sensitivity Troponin T (hs-TnT, Roche assay) measurements. All tests were conducted at baseline, 1 month, and 3 months following commencement of anthracyclines. 6 normal volunteers (3 male, 3 female) underwent the same CMR protocol. For oedema imaging, a triple inversion recovery fast-spin echo sequence (short-TI inversion recovery) was used in 3 short-axis views of the left ventricle. For assessment of myocardial oedema, the ratio of mean signal intensity (SI) of the myocardium was compared to that of skeletal muscle.

## Results

In the study patients, the mean CMR LV end-systolic volume index (LVESVI) increased from baseline of 17.8 ± 6.2 to 20.3 ± 5.9 mL/m2 (p<0.001) at 1 month and remained significantly elevated at 3 months (22.7 ± 5.6 mL/m2;p<0.001). Similarly, the end-diastolic volume index (LVEDVI) increased from 62.9 ± 11.6 mL/m2 at 1 month to 66.4 ± 11.8(p<0.000) at 3 months . Echocardiographic measurement of global longitudinal strain (GLS) showed no significant change at 1 month, however decreased in absolute magnitude from -21.4 ± 3% baseline to -19.5 ± 1% (p ≤ 0.001) at 3 months. Left ventricular diastolic function did not change significantly in study patients, either 1 or 3 month post chemotherapy. BNP did not correlate significantly with observed changes in LVEDV, LVESV and GLS. Hs-TnT did not change at 1 month but increased from 3.8 ± 1.6 to 10.7 ± 9.8 at 3 months (p ≤ 0.003). Although there was no difference between baseline values of T2 ratio between normal volunteers and study patients, 15/26 (58%) of patients had an abnormal T2 signal (SI increase to > 1.9 as specified by Lake Louis criteria) in one or more short axis slices post chemotherapy. There was no new late gadolinium hyperenhancement to suggest focal myocardial necrosis/fibrosis in any patient following therapy.

## Conclusions

Our data suggest that there are subtle functional changes in the LV myocardium early post anthracycline chemotherapy that can be detected by both CMR and advanced echocardiographic techniques. This is likely mediated by myocardial inflammation (without necrosis). These findings may be a basis for development of early predictors of cardiac damage and earlier intervention/preventative strategies.

## Funding

This study has received a grant from FMC Foundation, Adelaide, Australia.

